# Clinical Course and Outcome in Children with Congenital Hyperinsulinism

**DOI:** 10.34172/aim.2022.70

**Published:** 2022-07-01

**Authors:** Hedyeh Saneifard, Elham Hajihashemi, Minoo Fallahi

**Affiliations:** ^1^Shahid Beheshti University of Medical Sciences, Tehran, Iran; ^2^Pediatric Department, Shahid Beheshti University of Medical Sciences, Tehran, Iran; ^3^Neonatal Health Research Center, Research Institute for Children Health, Shahid Beheshti University of Medical Sciences, Tehran, Iran

**Keywords:** Congenital Hyperinsulinism, Hydrocortisone, Hypoglycemia

## Abstract

**Background:**

Hyperinsulinism is the most common cause of persistent or recurrent neonatal hypoglycemia that may result in neurological deficits. The treatment goal in these patients is prevention of hypoglycemia to decrease mortality and morbidity. This study was done to determine the clinical course and outcome in children with congenital hyperinsulinism (CHI) referring to Mofid Children’s Hospital from 2011 to 2017.

**Methods:**

This study was done on 22 children with CHI referring to Mofid Children’s Hospital from 2011 to 2017. The demographic, perinatal, clinical, laboratory, imaging, pharmacological, treatment and follow up data of these children were collected and analyzed.

**Results:**

Among 22 children with CHI, the mortality rate was higher among those who received hydrocortisone versus those who did not receive hydrocortisone (46% versus 40%).

**Conclusion:**

According to the results of this study, hydrocortisone had a negative impact on the outcomes of these children, which is important in the management of hypoglycemia. The clinical course and outcome of children with CHI was better with medical compared to surgical treatment.

## Introduction

 Congenitalhyperinsulinism (CHI) was introduced by MacQuarries et al in 1954 as idiopathic hypoglycemia of infancy.^1^ Other names such as persistent hyperinsulinemic hypoglycemia of infancy, islet dysregulation syndrome, leucine-sensitive hypoglycemia and nesidioblastosis have been proposed for this condition.^2^ The background mechanisms including genetic etiologies are not fully understood for many cases of CHI.^3^ One of the causes of hypoglycemia in infancy and childhood is HI which is accompanied by high mortality and morbidity rate and leads to irreversible neurological damage.^4-9^ In the majority of countries, the disease rate varies from one in 50 000 to one in 25 000 births. HI is diagnosed in nearly 60% of neonates in the first month of life. The remaining cases are diagnosed in the first year of life. It is more common in male neonates. Prompt treatment is associated with less brain injury and better outcomes. Mutations in the ABCC8 and KCNJ11 genes are the most common causes of HI, responsible for about 40%–45% of cases. Mutations in five other genes are seen in 5%–10% of cases and the genetic cause is unknown in 45%–55% of cases.^10^

 Complications of hypoglycemia include permanent brain damage, developmental delay and seizure.^11^ Hence, the therapeutic goal in these patients should be prevention of hypoglycemia to prevent mortality and morbidity. There are multiple medical and surgical therapeutic modalities that affect the clinical course and outcomes. Assessment of these therapies would help to determine the best therapeutic modality to achieve optimal outcome and decrease complications. Considering the lack of such studies in Iran, this study was done to determine the clinical course and outcome of children with CHI referring to Mofid Children’s Hospital from 2011 to 2017.

## Patients and Methods

 This is a case-series study conducted on 22 children hospitalized with a diagnosis of CHI from 2011 to 2018 at Mofid Children’s Hospital, Tehran, Iran. The cases with incomplete data and loss to follow-up were excluded. Two patients were lost to follow-up and their data were not included for statistical analysis

 This study was approved by the ethical committee of Shahid Beheshti University of Medical Sciences (891–1396) and the parents of children signed informed consent form for the study. This study had two stages. In the initial stage, data about children with CHI were extracted from medical records. These data included demographic and perinatal data (birth weight, gestational age, sex, gestational diabetes mellitus history, related parents, family history), clinical data (seizure, hypotonia, apnea, lethargy), treatment measures at the time of hospitalization (history of surgery, times of surgery, age at first and second surgery, hospital stay in days, death, age at death and medical treatments).

 In the second stage of this study, the patients were visited in the pediatric clinic and follow-up data were collected using a questionnaire from January 3, 2018 to February 20, 2018. The follow-up data included follow-up duration, death,neurodevelopmental disorder, seizure after discharge, diabetes, exocrine pancreatic dysfunction, diazoxide dose, octreotide dose, need for continuation of hydrocortisone and anti-convulsants, adverse effect of drugs and need for insulin.

 Data were analyzed using SPSS version 16.0. The utilized tests were Mann-Whitney U test, chi-square, and Fisher. Level of significance was 0.05.

## Results

 The demographic, perinatal and clinical data of the children with CHI are demonstrated in Tables 1 and 2. Data related to treatment measures at the time of hospitalization and follow-up data are shown in Tables 3 and 4.

**Table 1:** Demographic and Perinatal Data of Children with Congenital Hyperinsulinism (n = 22)

**Case**	**Birth Weight (g) Mean **	**Gender**	**GDM History**	**Consanguineous Parents**	**FH**	**Gestational age (wk)**	**Serum Insulin level ( μg/mL)**
1	4800	M	+			38	24
2	4560	F				35	24.9
3	3800	M				38	29
4	4200	M		+		39	124
5	5000	M				36	25
6	3200	M		+		38	2.8
7	3750	M		+		36	26
8	5100	F		+		39	51
9	3300	F		+	+	33	26
10	4900	F		+	+	38	88
11	4200	F	+	+	+	38	23
12	3000	M		+	+	39	52
13	2700	F	+			34	23
14	3450	F	+	+		35	28
15	3500	F	+	+		38	5.8
16	2850	F		+		36	8.5
17	3700	F		+		38	33
18	4500	F		+		39	31
19	3500	M		+		38	8.5
20	3050	F		+		34	24
21	3600	M		+		34	41
22	5400	M		+		35	29

**Table 2:** Clinical Data of the Children with Congenital Hyperinsulinism (n = 22)

**Case**	**Seizure**	**Hypotonia**	**Apnea**	**Lethargy**
1	+			
2	+			
3	+	+		
4	+			
5	+			
6	+			
7	+		+	
8	+	+	+	
9	+			
10	+			+
11	+	+		+
12				
13	+			
14	+			
15	+			
16	+		+	
17	+			
18	+			
19	+			
20	+			
21				
22	+			

**Table 3:** Data Related to Treatment Measures at the Time of Hospitalization of the Children with Congenital Hyperinsulinism (n = 22)

**Case**	**History of Surgery**	**Times of Surgery**	**Age at First Surgery** **(day)**	**Age at Second Surgery** **(mon)**	**Hospital Stay (days) **	**Death**	**Age at Death (days)**	**Diazoxide Dose on Admission** **(mg/kg/d)**	**Octreotide Dose on Admission** **(μg/kg/d)**	**Hydrocortisone Use on Admission**	**Anti-convulsant Use on Admission**
1	+	1	60		52			20	20		
2	+	1	23		20	+	60	25	20	+	+
3					12			25	20		+
4					30			25	20		+
5	+	1	35		50	+	49	25	20	+	+
6					7			20			+
7	+	1	20		150	+	165	25	20	+	+
8	+	1	71		92	+	910	25	20	+	+
9	+	1	22		43	+	60	25	20	+	+
10	+	1	9		97	+	912	25	20	+	+
11	+	1	27		45			25	20	+	+
12					6			3			
13	+	1	100		112			25	20		
14					50			25	20	+	+
15					36			25	20	+	
16					24			25	20	+	+
17					41			25	20		
18	+	2	53	11	83			25	20	+	+
19	+	1	24		50			25	20		
20					15			20		+	+
21	+	1	33		62			20	20		
22	+	1	23		38			25	20		+

NDD, neurodevelopmental disorder.

**Table 4:** Follow-up Data of the Children with Congenital Hyperinsulinism (n = 22)

**Case**	**Follow-Up Duration** **(mon)**	**Death**	**NDD**	**Seizure After Discharge**	**Diabetes**	**Exocrine Pancreatic Dysfunction**	**Diazoxide Dose** **(mg/kg/d)**	**Octreotide Dose** **(μg/kg/d)**	**Need for Hydrocortisone**	**Need for Anti-convulsant**	**Adverse Effect ** **of Drugs**	**Need for Insulin **
1	34											
2	2	+					25	20	+	+		
3	90						1			+		
4	11						2			+		
5	1.5	+					30	12	+	+		
6	84		+	+			7					
7	5.5	+	+	+			25	20	+	+		
8	30	+	+	+			25	20				
9	2	+					25	25				
10	30	+	+	+	+	+				+		+
11	12									+		
12	108						3					
13	84				+	+						+
14	96		+	+						+	+	
15	1						15	15	+			
16	35			+				25		+	+	
17	6						20					
18	12						7		+	+		
19	2		+	+			20	25		+		
20	84						15			+		
21	36											
22	0.5							20		+		

 The demographic and perinatal data showed that the mean (*standard deviation) *birth weight was 3911.8 (795.59) grams. Also, 63.6% were delivered by cesarean section. Ten of 22 (45.4%) patients were male. History of gestational diabetes mellitus was positive in five patients (22.7%). Seventeen patients (77.2%) had consanguineously related parents and family history was positive in four patients (18%). The mean (*standard deviation) *level of serum insulin was 32.04 (27.6).* There was no correlation between *serum *insulin level and mortality rate (P value: 0.28) or need for surgery (P value: 0.82)*.

 Clinical data indicated that 20 of 22 (90%) patients had seizure and 3 patients had hypotonia (13.6%). Apnea and lethargy were reported in three (13.6%) and two patients (9%), respectively.

 Data related to treatment measures at the time of hospitalization showed that 13 of 22 (59%) patients had a history of surgery. Times of surgery was once in 12 (54.4%) patients and twice in only one patient (4.5%). The mean (standard deviation) age at first surgery was 38.46 (25.59) days and age at second surgery was 11 months. The mean (standard deviation) number of days of hospital stay was 50.6 (36.4) days. Six patients (27.2%) died and the mean (standard deviation) age at death for these patients was 7.3 (11.1) months. The mean (standard deviation) diazoxide dose in hospital was 23 (4.9) mg/kg/d. The mean (standard deviation) octreotide dose was 19 (7.02) μg/kg/d. Twelve of 22 patients (54.5%) used hydrocortisone and 15 of 22 patients (68.1%) used anti-convulsant drugs. According to the results of Mann–Whitney test, there was a significant difference between children who *received and those who did not* receive hydrocortisone regarding mortality rate (*P* value: 0.015) and hydrocortisone doubled the mortality rate.

 Follow-up data indicated that the mean follow-up duration was 36.5 (SD: 37) months. Neurodevelopmental disorders were seen in six patients (27.2%). Seizure after discharge was seen in 7 of 22 patients (31.8%). In two patients (9.1%), diabetes developed and two patients (9.1%) had exocrine pancreatic disorder. The mean diazoxide dose was 16 (sd:10) mg/kg/d, and fourteen patients (63.6%) were taking diazoxide. The mean octreotide dose was 21 (sd:4.25) μg/kg/d and nine patients (40.9%) were using octreotide. Hydrocortisone was used in five patients (22.7%). Anti-convulsant drugs were needed in 13 patients (59%). Drug adverse effects were seen in 9% of patients. Insulin was needed in two patients (9%).

## Discussion

 CHI is a genetic clinicopathological disorder about which there is little information available. Regarding the important side effects of hypoglycemia in children with CHI, the choice of appropriate therapeutic method is important. Hence, assessment of the clinical course and outcome is essential to find the best treatment option leading to better prognosis in patients. *To the best of the authors’ knowledge, no research has evaluated the effect of hydrocortisone on mortality rate in *children *with CHI*.

*The protocol used for starting and continuing the drugs was as follows: At first, glucose infusion was started at 10–15 mg/kg/min*.* If hypoglycemia continued, the glucose infusion was increased to 20 mg/kg/min via a central catheter or the umbilical vein. Oral diazoxide was used at the initial dose of 5–15 mg/kg/d and increased to 20–25 mg/kg/d if necessary*.* Octreotide was used in case of non-responsiveness to diazoxide, starting at an initial dose of 5–15 *μg/kg/d*. In cases of unresponsiveness, the dose was slowly increased to 20–25 *μg/kg/d*. In cases suspicious for cortisol deficiency and in retractable hypoglycemia, hydrocortisone was started and surgical consultation was requested*.The results of our study indicated that the clinical course and outcome of children with CHI under medical therapy was better compared with surgical treatment. According to the result of Mann–Whitney test, there was a significant difference between children who *received and those who did not* receive hydrocortisone regarding mortality rate (P value: 0.015) and hydrocortisone doubled the mortality rate.

 Lord et al evaluated the clinical symptoms, treatment, and outcomes in cases with diffuse and focal HI in a retrospective study between 2004 -2014. They found that focal cases were more probable to experience delayed diagnosis and hypoglycemic seizures, but the majority of them were cured with surgery.^12^ In their study, the diffuse cases were diagnosed earlier, but had more fluctuation in blood glucose levels; however, in our study, the differentiation between focal and diffuse types was not done.

 The study by Arya et al conducted on 300 patients with diffuse CHI demonstrated that 45 cases had no therapeutic response to medical therapy and underwent near-total pancreatectomy. The prevalence rate of insulin-dependent diabetes was 96% after 11 years from surgery and there was exocrine pancreatic disorder in 72%.^13^ In the current study, there was insulin-dependent diabetes in two patients (15.3%) and exocrine pancreatic disorder in two patients (15.3%). It should be mentioned that the follow-up time in our study was shorter than previous studies. Type of surgery had no impact on outcomes.

 The study done by Beltrand et al assessed the long-term metabolic status in patients with CHI after pancreatectomy in 105 children. The disease developed in 59% of cases with symptoms ranging from asymptomatic to mild hypoglycemia. They concluded that in patients with focal CHI, hypoglycemia is treated after surgery but the response is variable in diffuse cases.^14^ The prevalence of insulin-dependent diabetes in early adolescence is very high but, in our study, surgical therapy was associated with poorer prognosis and increased mortality rate.

 In another study conducted by Barthlen et al on 103 cases with CHI, they showed that hypoglycemia persisted after pancreatectomy in 36% of cases. Thirty-one percent of patients had hyperglycemia and diabetes mellitus but in our study, 9% had hyperglycemia and 3 out of 13 patients who underwent pancreatectomy, had hypoglycemia that needed treatment. On follow-up, one patient was treated with diazoxide (15 mg/kg), one patient with octreotide (20 μg/kg) and one patient required two drugs for treatment.^15^

 The study by Palladino et al on 250 patients with CHI showed that hypoglycemia persisted in 50% and also 25% developed hyperglycemia that required insulin therapy and only 25% were euglycemic. The results of their study showed that glycemic control after pancreatectomy is easier. In our study, 72.7% remained hypoglycemic while 22.7% were euglycemic.^11^

 Cherian andAbduljabbar assessed 10 patients with diffuse CHI and all had diabetes mellitus after pancreatectomy. Among them, three cases had diabetes immediately after surgery and the mean time to develop diabetes in the other cases was eight years. However, in our study, the success rate was higher and the rate of diabetes was lower and only 9% used insulin after discharge. Nevertheless, it should be mentioned that the follow-up time in our study was shorter than previous studies.^16^

 Salomon-Estebanez et al assessed the result of conservative therapy in CHI cases and they found that it was cured in the majority of cases. However, cure was not achieved in all cases but the decrease in severity was a promising point on follow-up as seen in our study.^17^

 Demirbilek et al assessed 28 patients with CHI and found that a transient increase in hepatic enzymes and asymptomatic pathology in gallbladder are the most common side effects of octereotide.^18^ In our study, the side effects of diazoxide were seen in 9% but we did not observe any adverse effects of octreotide. In the follow-up of the current study, only four patients were under treatment with octreotide, three of whom received treatment for 1-2 months and only one case required long-term octreotide therapy.

 In another study done by Glaser et al, they assessed the long-term effects of somatostatin analogues for treatment of HI in children and found that response to this therapy is variable and unpredictable. Intravenous glucose infusion was continued in two cases. In two other cases, it was discontinued by continuous nasogastric feeding and in five remaining cases, the nutritional pattern was normalized.^19^

 It should be mentioned that the present study had some limitations which should be considered.One limitation of this study was lack of genetic assessment in patients and parents. Lack of differentiation between focal and diffuse types of the disease was another limitation. In this study, the rate of diabetes and exocrine pancreatic insufficiency was lower than similar studies that may be due to the short follow-up duration of some patients in the current study. Hence, it is suggested that future studies should be done with consideration of these limitations and also on larger sample populations and by multi-center sampling.

 In conclusion, according to the results of our study, it is concluded that the clinical course and outcome in children with CHI under medical therapy is better than cases for whom surgical treatment was done. Hydrocortisone had a negative effect on the outcome of patients that may be an important point in management of hypoglycemia.
